# Managing Hytrosavirus Infections in *Glossina pallidipes* Colonies: Feeding Regime Affects the Prevalence of Salivary Gland Hypertrophy Syndrome

**DOI:** 10.1371/journal.pone.0061875

**Published:** 2013-05-07

**Authors:** Adly M. M. Abd-Alla, Henry M. Kariithi, Abdul Hasim Mohamed, Edgardo Lapiz, Andrew G. Parker, Marc J. B. Vreysen

**Affiliations:** 1 Insect Pest Control Laboratory, Joint FAO/IAEA Programme of Nuclear Techniques in Food and Agriculture, Vienna, Austria; 2 Laboratory of Virology, Wageningen University, Wageningen, The Netherlands; Wuhan Bioengineering Institute, China

## Abstract

Many species of tsetse flies are infected by a virus that causes salivary gland hypertrophy (SGH) syndrome and the virus isolated from *Glossina pallidipes* (GpSGHV) has recently been sequenced. Flies with SGH have a reduced fecundity and fertility. Due to the deleterious impact of SGHV on *G. pallidipes* colonies, several approaches were investigated to develop a virus management strategy. Horizontal virus transmission is the major cause of the high prevalence of the GpSGHV in tsetse colonies. Implementation of a “clean feeding” regime (fresh blood offered to each set of flies so that there is only one feed per membrane), instead of the regular feeding regime (several successive feeds per membrane), was among the proposed approaches to reduce GpSGHV infections. However, due to the absence of disposable feeding equipment (feeding trays and silicone membranes), the implementation of a clean feeding approach remains economically difficult. We developed a new clean feeding approach applicable to large-scale tsetse production facilities using existing resources. The results indicate that implementing this approach is feasible and leads to a significant reduction in virus load from 10^9^ virus copies in regular colonies to an average of 10^2.5^ and eliminates the SGH syndrome from clean feeding colonies by28 months post implementation of this approach. The clean feeding approach also reduced the virus load from an average of 10^8^ virus copy numbers to an average of 10^3^ virus copies and SGH prevalence of 10% to 4% in flies fed after the clean fed colony. Taken together, these data indicate that the clean feeding approach is applicable in large-scale *G. pallidipes* production facilities and eliminates the deleterious effects of the virus and the SGH syndrome in these colonies.

## Introduction

Tsetse flies (*Glossina* spp.) are the only vectors of two debilitating diseases in Africa, sleeping sickness in humans (human African trypanosomosis, HAT) and the cattle disease nagana (or African animal trypanosomosis, AAT) [1], [2]. Nagana, and in certain areas also sleeping sickness, has been a major obstacle to sub-Saharan African rural development and a severe constraint to agricultural production [3]. Due to the lack of effective vaccines and inexpensive drugs for the treatment of HAT, and the development of resistance of the AAT parasites to available trypanocidal drugs [4], vector control remains the most efficient strategy for the sustainable management of these diseases [3], [5].

The successful eradication of *Glossina austeni* from Unguja Island, United Republic of Tanzania, was achieved using an area-wide integrated pest management (AW-IPM) approach [6] including the release of sterile male flies [7]. Based on this success, programmes were developed to apply this approach on the African mainland and, in 1996, the Government of Ethiopia embarked on such a programme with the aim of creating a zone free of *Glossina pallidipes* in the Southern Rift Valley of Ethiopia [8], [9]. Unlike the relatively easy establishment and maintenance of a *G. austeni* colony in Tanzania, the establishment and expansion of *G. pallidipes* colonies for mass production of sterile male flies proved to be difficult and they collapsed several times due to the deleterious effect of high infection rates with the salivary gland hypertrophy virus [10]–[13].

The *G. pallidipes* salivary gland hypertrophy virus (GpSGHV) is a member of Glossinavirus species belonging to the *Hytrosaviridae* family [14]. This family consists of circular double stranded DNA viruses that inhibit reproduction by suppressing vitellogenesis, causing testicular aberrations, and/or disrupting mating behaviour [15].

In wild tsetse populations mother to offspring transmission, either trans-ovum or through infected milk glands, is thought to be the most likely mode of transmission of the virus [16]–[18]. In laboratory-maintained flies horizontal transmission during blood feeding using an *in vitro* membrane feeding system [19] was found to be the most important route of virus transmission, as each tray of blood may be used to feed up to ten successive sets of fly cages [12]. To develop GpSGHV management strategies several studies were conducted on various aspects of the biology of the virus including the complete sequencing of the virus genome [11], establishment of virus dynamics in tsetse colonies [12], assessment of virus prevalence in wild tsetse populations [20], assessment of the impact of the virus on tsetse mating behaviour [21] and a determination of the protein composition and the localization of the proteins in the virus ultrastructure [22], [23]. Following these basic studies, several virus management approaches were proposed as recently reviewed [13], [24]. Although these management approaches were effective in reducing the SGH prevalence in laboratory *G. pallididpes* colonies, they entailed additional cost.

Historically, colony tsetse flies were initially fed on live animals [25]–[27], but this technique faced many challenges to produce the required number of high quality sterile males. Feeding techniques were significantly improved with the development of an *in vitro* feeding system whereby blood was offered under a silicone membrane [28]–[30], [30]–[32]. The feeding system (Mews et al [33] was optimized and adopted for the mass-rearing of most tsetse species [19], [34]–[37]. To reduce operational costs, several rounds of fly cages are normally fed in sequence on the same membrane [19]. This feeding regime provides excellent conditions for horizontal virus transmission as the infected flies release large numbers of virus particles via the saliva immediately prior to blood uptake, which are infectious to healthy flies feeding on the same membranes [12]. Due to the high fly densities in mass-rearing facilities, the membrane feeding system favoured efficient virus transmission leading to a high prevalence of SGH syndrome which has been implicated in the collapse of three *G. pallidipes* colonies (two in Seibersdorf in 1987 and 2001 and one in Ethiopia in 2012).

This paper presents data on the implementation of a protocol to eliminate the SGH syndrome and to significantly reduce GpSGHV infections in *G. pallidipes* colonies without additional resources. Based on the data presented here, a modified protocol for the management of large-scale tsetse production facilities is proposed.

## Materials and Methods

### 1. Tsetse Fly Colonies

Fly material from a *G. pallidipes* colony maintained since 1987 on an *in vitro* feeding system at the Insect Pest Control Laboratory, Seibersdorf, Austria [19], [37] and originating from pupae collected near Tororo, Uganda in 1975 (Seibersdorf Tororo colony) was used in the study.,For baseline infection level comparison, results from four other untreated colonies are also presented: 1) A colony with the same origin as the previous colony established at the Institute of Zoology, Slovak Academy of Sciences, Bratislava, Slovakia (Bratislava Tororo colony), 2) a colony from the same origin as the previous colonies and maintained at the Tsetse Fly Rearing and Irradiation Center, Kality, Addis Ababa, Ethiopia (Kality Tororo colony), 3) a colony established at the Tsetse Fly Rearing and Irradiation Center, Kality, Addis Ababa, Ethiopia derived from pupae collected near Arba Minch, Ethiopia, between 1999 and 2001 (Kality Arba Minch old colony) and 4) a colony from the same origin as for colony 4, and established at Kality in the period 2008 to 2010 (Kality Arba Minch new colony).

### 2. Evaluation of SGH Prevalence in *G. pallidipes* Colonies Maintained at Different Tsetse Production Facilities

To monitor the prevalence of the SGH syndrome in the five *G. pallidipes* colonies outlined above, male and female flies of different ages were randomly selected from the five colonies, in 2010, 2011 and 2012. A total of 1,326 flies were dissected from the colonies at different ages, weekly units, and different days (2–110) post emergence (**Table 1**). The flies were anesthetized at −20°C for 3–5 min, placed on ice, dissected, and the status of the salivary glands recorded.

**Table 1:** Total numbers of randomly selected flies dissected from and prevalence of SGH syndrome in the five *G. pallidipes* fly colonies used in this study.

Colony Name	Total no. of dissected flies	No. of SGH^+^ flies	% of SGH^+^ flies
Seibersdorf Tororo	244	9	3.69^b,c^
Bratislava Tororo	309	9	2.91^c^
Kality Tororo	322	32	8.77^b^
Kality Arba Minch new	185	9	4.45^b,c^
Kality Arba Minch old	266	195	73.84^a^

Values followed by the same letter do not differ significantly (*P*>0.05).

### 3. Blood Feeding and Establishment of Clean Feeding Regimes

**Figure 1:**
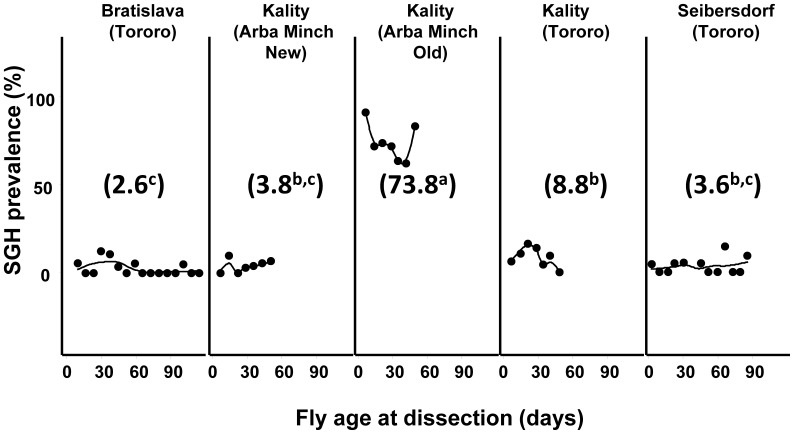
Prevalence of SGH in different tsetse colonies of *G. pallidipes.* The flies were randomly selected at different time points from the different colonies and dissected to determine status of the salivary glands. Numbers between brackets are the mean SGH prevalence percentage. The line is the smoothed regression.

Two protocols for feeding the Seibersdorf Tororo flies were used: 1) the standard membrane feeding protocol [19], whereby successive cages with flies were offered a blood meal on the same membrane, and 2) a “clean blood feeding protocol” (hereafter denoted as “clean feeding”), whereby each cage of flies was provided with new, sterile defibrinated cow blood at each meal [19] (**Figure 1**). The clean feeding protocol was used to prevent the flies from picking up the virus from the blood already used for feeding previous cages. To implement the clean feeding protocol in the large-scale colony, newly-emerged (teneral) *G. pallidipes* flies from the regular Seibersdorf Tororo colony were offered a clean blood meal and thereafter these flies and their progeny were always the first to be fed on fresh, clean blood during the entire period of the experiment. This colony was denoted as “clean feeding colony 1″ (CFC-1), and was expanded by addition of teneral flies emerging from the CFC-1 parents until the maximum number of cages that could be fed first (during one round of feeding) on available feeding trays was attained. Subsequently, when the maximum number of cages for the CFC-1 colony was attained, the excess flies from the CFC-1 progeny were fed on the same membrane as a second feeding round after feeding the CFC-1 colony. This second-round fed group of flies was denoted as “clean feeding colony 2″ (CFC-2), and was always maintained on a second feeding round after feeding the CFC-1 colony throughout the entire experimental period. During the establishment of the CFC-1 and CFC-2, the regular colony was always fed on the same membranes used to feed CFC-1 and -2 (at the third and subsequent feeding rounds), and was denoted as “normal feeding colony” (NFC).

### 4. Impact of Clean Feeding Regime on SGH Prevalence

To assess the impact of the clean feeding regime on the prevalence of SGH in Seibersdorf Tororo *G. pallidipes* colonies, 20 flies were randomly selected for dissection from each of twelve or thirteen different weekly units from the CFC-1, CFC-2 and NFC colonies at 6, 11 and 28 months post implementation of the clean feeding regime. The status of the salivary glands was recorded.

### 5. Impact of Clean Feeding Regime on GpSGHV Copy Numbers

To determine the initial levels of GpSGHV in the Seibersdorf Tororo *G. pallidipes* colonies, eight flies were randomly selected from six different weekly units from the main colony that was used to initiate the clean feeding system. To determine the impact of the clean feeding regime on virus loads, eight flies were also sampled from each of twelve units from the CFC-1, CFC-2 and NFC colonies after 28 months of implementation of the clean feeding regime. Total DNA was extracted from the sampled individual flies using DNeasy kit (Qiagen) according to the supplier’s instructions. Quantitative PCR (qPCR) was carried out on the extracted DNA using the primers and amplification conditions previously described [38].

### 6. Statistical Analysis

Differences in the log virus copy numbers were assessed by analysis of variance (ANOVAR) and individual treatment means were compared with the Tukey-Kramer HSD test [39]. Analysis was performed using Excel® 13 (Microsoft Corp.), RExcel [40] and R [41]. The graphics were created using the ggplot2 package in R. The smoothed regression line was calculated using the default local polynomial regression fit (Loess) in the ggplot2 library.

## Results

### 1. Prevalence of SGH in Different *G. pallidipes* Colonies

The results of the dissection presented in **Figure 1** shows that the SGH prevalence varied significantly (df = 4, 44; F = 234.78; *P*<0.00001) between the five different colonies. With the exception of the Kality Arba Minch old colony which showed a high SGH prevalence of 74%, SGH prevalence in the other *G. pallidipes* colonies was less than 10% regardless of fly age **(** **Figure 1** and **Table 1**). The regression of SGH prevalence on fly age was not significant (df = 1, 40; F = 3.076; *P* = 0.087).

### 2. Feasibility of Establishing a Clean Feeding Colony with Existing Resources

**Figure 2:**
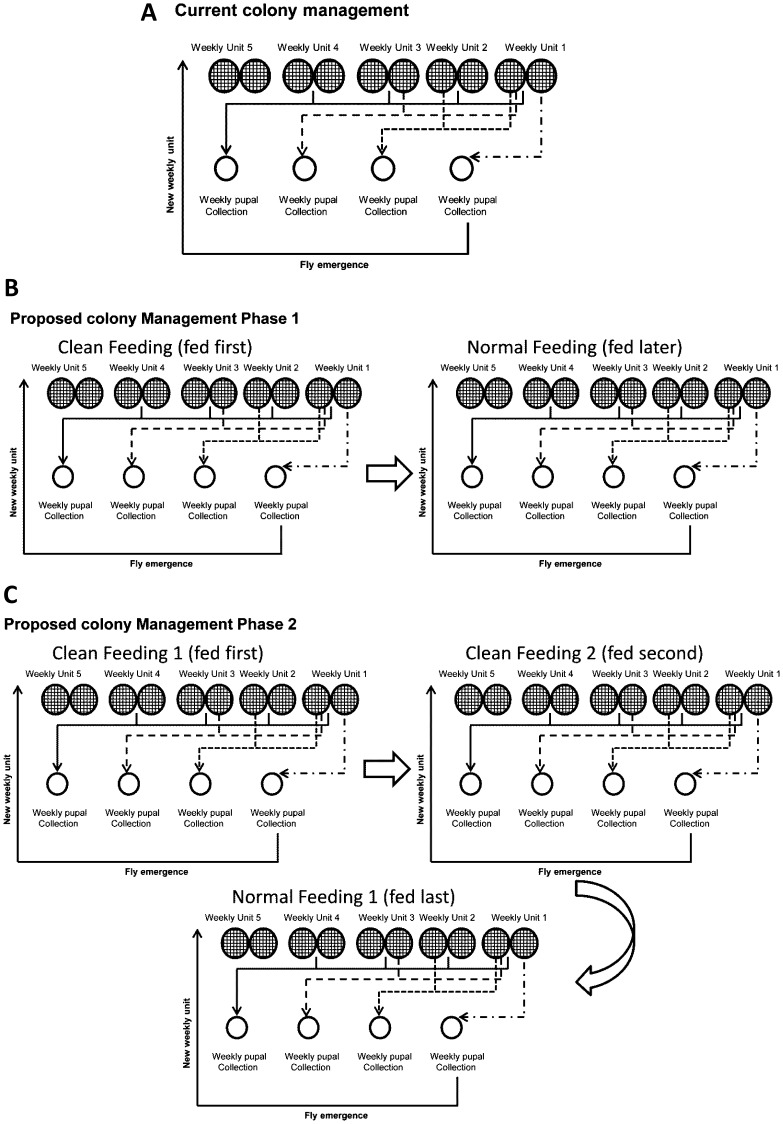
Handling, feeding and management of tsetse colony. (A) Regular *in vitro* membrane feeding system, (B) intermediate phase, and (C) final phase of the clean feeding system.

The main Seibersdorf Tororo *G. pallidipes* colony (totalling ∼12,000 flies) was maintained to provide pupae for establishing the clean feeding colonies using existing resources (i.e. no increase in number of staff, feeding trays and silicone membranes) **(** **Figure 2A** **)**. With the available 12 sets of trays and silicone membranes, each able to feed 4 fly cages, the size of the CFC-1 and CFC-2 colonies was limited to 48 cages each, each colony containing on average 2,500 male and female flies (the standard cage initially contains 60 females and 15 males). Initiation of the clean feeding colonies started with feeding teneral flies (4 cages/week) emerging from the main colony. Twelve weeks after starting the clean feeding, the CFC-1 colony reached 48 cages and a higher number of teneral flies emerged from this colony than the number required to maintain the colony size stable **(** **Figure 2B** **)**. Therefore, excess teneral flies from CFC-1 were fed on the same membrane to establish CFC-2. Similarly, the CFC-2 colony reached 48 cages after 12 weeks by adding 4 cages of teneral flies each week. As a result of implementing the clean feeding protocol, the main Seibersdorf Tororo colony (∼10,000) was progressively replaced by the three colonies, i.e. the CFC-1 (∼2,500 flies), CFC-2 (∼2,500) and NFC (∼ 5,000 flies) **(** **Figure 2C**).

### 3. Impact of Clean Feeding Regime on SGH Prevalence in the Seibersdorf Tororo Colonies

**Figure 3:**
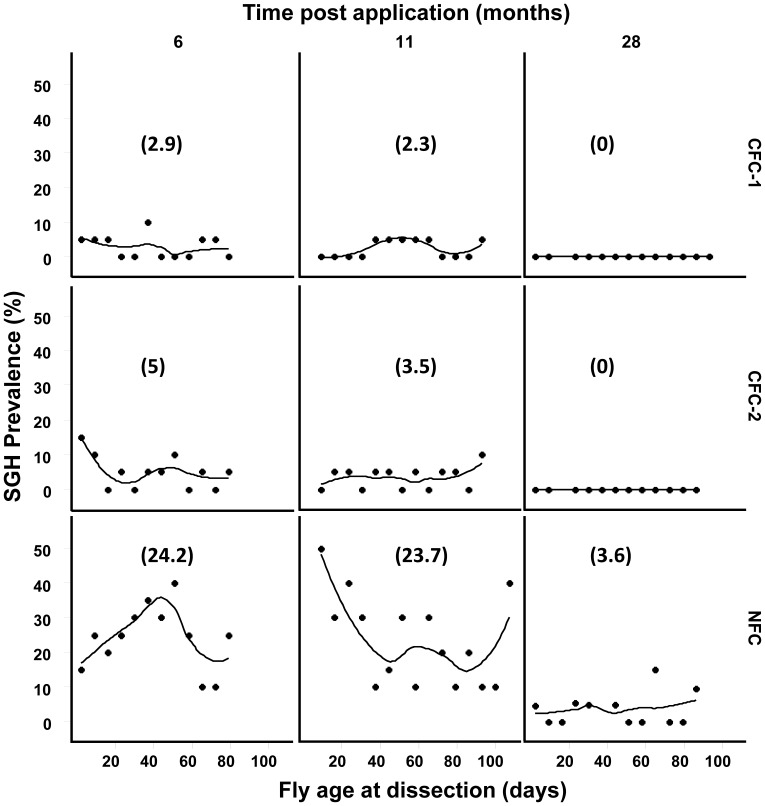
Effect of long-term clean feeding system on SGH prevalence in the Seibersdorf Tororo *G. pallidipes* colonies by fly dissection. The flies were randomly selected at different time points from the different colonies after implementation of the clean feeding system and dissected to determine status of the salivary glands. Numbers between brackets are the mean of SGH prevalence percentage; lines are Loess smoothed regressions.

The dissection results presented in **Figure 3** show that the prevalence of the SGH syndrome in the CFC-1 was significantly reduced over the experimental period. Within 6 months of the implementation of the clean feeding regime, the SGH prevalence was reduced from 5–10% (the normal average prevalence in the Seibersdorf Tororo colony before treatment) to an average of 2.9%, irrespective of the age of the flies. After 11 months, the prevalence was further reduced to 2.3%, and SGH was completely eliminated in CFC-1 after 28 months. A similar trend was observed in the CFC-2 colony (**Figure 3**). In the NFC colony, there was a temporary increase in SGH prevalence to 24% during the first 6 months (with some weekly units exhibiting a prevalence of up to 40%). After 11 months, the SGH prevalence in the NFC colony averaged 23.7%, (with an average prevalence of ∼ 50% in younger flies (0–20 days old)). This rise in SGH prevalence was presumably due to the NFC flies being exposed to virus in the blood from emergence giving greater time and opportunity for infection. It is not clear if teneral flies are more susceptible to infection than older flies. However, after 28 months of the experiment, the SGH prevalence in the NFC was significantly reduced to 3.6%This was much lower than the levels regularly seen in standard-fed *G. pallidipes* colonies. In addition, while the SGH prevalence in the NFC colony appeared to vary with fly age during the first year of the experimental period, the prevalence stabilized later irrespective of the age of the flies (**Figure 3**). It is presumed that the initial increase in SGH prevalence was due to the teneral flies of the NFC being exposed to virus from the CFC-1 and CFC-2 feeding before the virus level decreased in these colonies, and subsequently declined after the SGH prevalence in the CFCs had decreased leading to the low, stable age-specific rates observed in the NFC.

### 4. Impact of Clean Feeding Regime on the Virus Load in the Seibersdorf Tororo Colony

**Figure 4:**
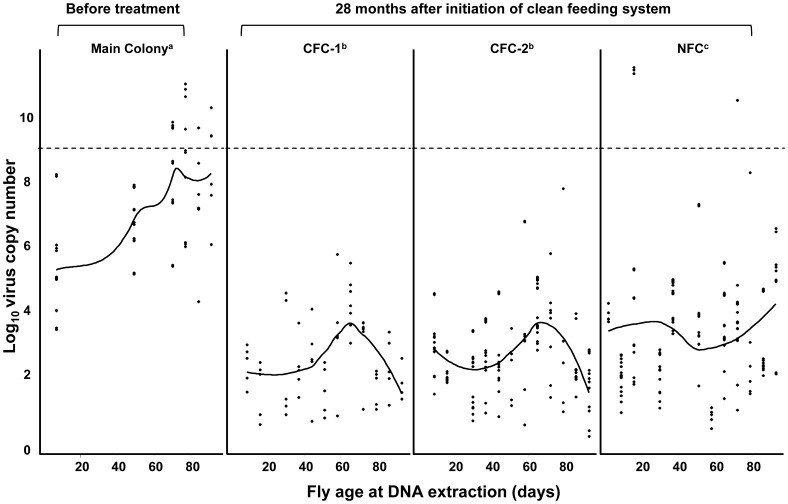
Effect of long-term clean feeding system on the prevalence of GpSGHV in the Seibersdorf Tororo *G. pallidipes* colonies. Virus load was determined by qPCR of the main colony before initiation of the clean feeding system and from the three colonies Clean feeding 1 (CFC-1), Clean feeding 2 (CFC-2) and normal feeding system (NFC) after 28 months of implementation of the clean feeding system. (---): threshold virus load correlated with SGH symptoms; solid lines are Loess smoothed regressions.

Quantification of the virus copy numbers using qPCR revealed a significantly lower virus load in the CFC-1, CFC-2 and NFC colonies (average of 10^2.4^, 10^2.7^ and 10^3.3^, respectively) after 28 months, as compared to the colony prior to the start of clean feeding where the virus load averaged 10^7.4^ copies, (df = 3, 676; F = 222.98; *P*<0.0001) (**Figure 4**). Interestingly, while the regression of virus load in the main Seibersdorf Tororo colony before the start of clean feeding on the age of the flies increased significantly (df = 1, 103; F = 50.504; *P*<0.0001), no significant correlation between virus load and fly age was found in CFC-1 and CFC-2. Also, there were no flies in the CFC-1 and CFC-2 colonies exhibiting virus levels normally associated with the SGH syndrome (i.e. ≥10^9^ virus copies). In addition, while the NFC colony showed a similar trend to the Seibersdorf Tororo colony before the start of clean feeding i.e. a general increase in virus loads with increasing age of the flies, the prevalence was significantly lower than that of the main colony, with only 2.2% (4/186) of the flies having a virus load that would be associated with the presence of the SGH syndrome (≥10^9^ copies).

## Discussion

Successful implementation of an AW-IPM programme with a sterile insect technique (SIT) component requires large-scale production and release of sterile males with acceptable quality in order to outcompete wild-type males for mating [6]. Unlike the majority of other insects with high reproductive rates such as fruit and screwworm flies, tsetse flies have very low productivity [42]. In addition, unlike tsetse flies which solely depend on blood, cheap and efficient larval diets have been developed for these highly productive insects which makes their large-scale production economically feasible [6]. The dependence of tsetse flies on blood alone as a nutritional source presents unique challenges for the successful implementation of programmes that include the release of sterile insects. Initial attempts to colonize tsetse flies used live host animals for fly feeding but this required the maintenance of a large number of the host animals, making the establishment and expansion of tsetse colonies difficult and expensive [25]–[27]. The development and implementation of an *in vitro* membrane feeding system [32] greatly contributed to the success of the program that eradicated a *G. austeni* population from Unguja Island, United Republic of Tanzania [7]. The creation of a sustainable area free of *G. austeni* on Unguja Island elicited interest in other African countries to use a similar integrated area-wide approach with an SIT component against other tsetse species on mainland Africa [8], [9].

Attempts to use the membrane feeding technique to upscale production in a *G. pallidipes* facility in Ethiopia proved to be extremely challenging due to the rapid spread of GpSGHV in the colonies [12]. As a result, the colonies became extinct within a few years of their establishments. To address this serious and prevailing problem, studies were initiated to better understand the virus and its transmission dynamics and to eventually develop virus management strategies that would enable sustainable maintenance and expansion of *G. pallidipes* colonies. These studies have included the application of an anti-viral drug to prevent replication [13], [24] and antibodies and RNAi to prevent infection. After demonstrating that horizontal virus transmission was facilitated through the regular membrane feeding system and the main cause of the deleterious effect on colony stability and productivity [12], [13], it was hypothesised that modifying the feeding regime might reduce the transmission risk and, thereby, reduce dependence on the anti-viral drug that could result in drug resistance. However, in the absence of inexpensive and disposable colony maintenance materials (feeding trays and silicone membranes), the implementation of the “clean feeding system” in a large scale production facility would be impractical, as this would considerably increase the net cost of the rearing of the flies destined for use in field releases.

The data presented in this paper has clearly demonstrated that a clean feeding regime can be efficiently applied within existing resources in tsetse mass-rearing facilities. Whereas traditional colony feeding entailed the feeding of up to 10 sets of fly cages in succession on the same membrane, the clean feeding system offers a fresh blood meal for each fly cage and this resulted in a significant reduction in GpSGHV levels and complete elimination of the SGH syndrome. In addition, in regular tsetse colonies pupae are collected on a weekly basis, which leads to mixing the pupae produced by the younger females (which are fed on clean blood) and those produced by older females (which are fed later on the blood potentially contaminated by previous feeding). Mixing the pupae eventually leads to production of teneral flies having varying but significantly higher virus loads, and in many cases the emerging teneral flies exhibit fully developed SGH syndrome **(** **Figure 1A** **)**. When teneral flies with high virus titres and/or with SGH are fed first on the clean blood, up to 10^9^ virus particles/fly were release into the blood, which are subsequently infectious to other “healthy” flies in the colonies. This increased the prevalence of the virus and SGH syndrome drastically, thus making tsetse mass-rearing difficult [12]. The rate at which the virus load and SGH prevalence increased in the colony was closely linked to the number of feeding cycles on the same membrane. By changing colony management through the application of a clean feeding system, this disadvantageous mixing of pupae was avoided as demonstrated by the results of the CFC-1, CFC-2 and NFC (**Figure 3**).

To implement a clean feeding regime in tsetse production facilities, the main colony could be subdivided over time into three (or more) completely independent colonies, with separate data recording of the parents and progeny to monitor colony performance **(** **Figure 2C** **)**. The principle aim would be to separate the pupae produced by the females fed first on clean blood from those produced by the females fed later on, which have the risk of picking up virus from the blood potentially contaminated by flies fed previously. This risk of contamination is clearly shown in **Figure 1**, where the teneral flies used at the initiation of the clean feeding regime have unknown virus load and are, therefore, able to release the virus during feeding. However, as demonstrated by the CFC-1, the virus load and the SGH prevalence gradually decreases to below detectable levels. As a consequence, the flies in this colony did not release large numbers of virus particles into the blood during feeding and hence the flies fed on the second feeding round (CFC-2) essentially ingested clean blood as did the CFC-1 colony. Since the CFC-2 colony eventually showed no detectable levels of SGH and therefore stopped releasing large numbers of virus particles into the blood, the teneral flies of the NFC were consequently also fed on clean blood first and successively the older flies. Due to the reduction in the colony size in the NFC (∼5000 flies) compared with the colony size before the clean feeding system (∼10000 flies), the number of feeds per membrane was reduced, which resulted in a reduction in virus transmission and SGH prevalence of the colony.

Taken together, the data presented in this paper demonstrate the effectiveness of clean feeding techniques to reduce the virus load and to remove the SGH syndrome in colonies of *G. pallidipes* leading to sustainable maintenance and expansion of the colony. The data also show that the implementation of the feeding regime is readily applicable in tsetse mass rearing facilities within existing resources (in terms of staff and equipment) and only requires changes to the colony handling and recording system, which only need minimal additional training of the staff, at no significant additional cost to the SIT program compared to the cost of other methods to manage the virus. The data presented in this article strongly supports the implementation of a clean feeding technique in large-scale tsetse production facilities in order to achieve sustainable GpSGHV control.
